# Chemo-free treatment of adult patients with Ph-positive acute lymphoblastic leukemia: latest updates from the 2023 ASH annual meeting

**DOI:** 10.1186/s13045-024-01539-4

**Published:** 2024-04-16

**Authors:** Ting Shi, Hong-Hu Zhu

**Affiliations:** 1 Department of Hematology, Beijing Chao-Yang Hospital, Capital Medical University, No. 8 Gongren Tiyuchang Nanlu, Chaoyang District, Beijing, 100020 China; 2 Chinese Institutes for Medical Research, Beijing, 100005 China

**Keywords:** Ph + ALL, Chemo-free, TKIs, Blinatumomab, Venetoclax

## Abstract

The chemo-free concept represents a new direction for managing adult patients with Ph-positive acute lymphoblastic leukemia (Ph + ALL). The tyrosine kinase inhibitors (TKIs), blinatumomab and venetoclax serve as the backbone of chemo-free regimens; several prospective studies involving these drugs have demonstrated high remission rates and promising, albeit short, survival outcomes. This review summarizes the latest updates on chemo-free regimens in the treatment of adult patients with Ph + ALL, presented at the 2023 ASH annual meeting.

## To the editor

**Fig. 1:**
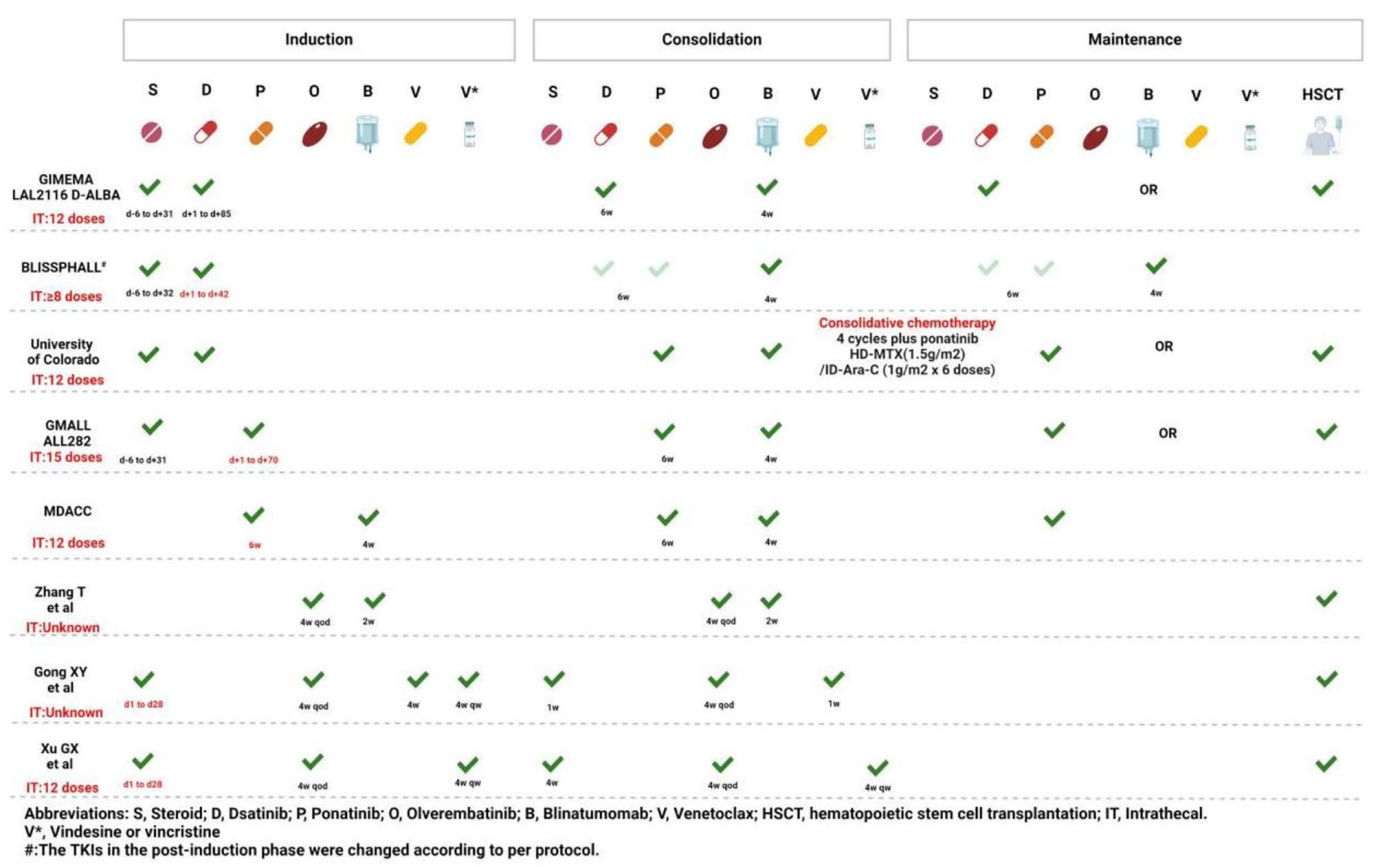
Study schema of each trial

Tyrosine kinase inhibitors (TKIs) combined with steroids results in high remission rates and minimal toxicity for adult patients with Ph-positive acute lymphoblastic leukemia (Ph + ALL); however, the cure rate remains unsatisfactory. Attempts are being made to design an optimal chemo-free regimen that incorporates new approaches, such as immunotherapy and small-molecule agents. This review summarizes the latest updates on chemo-free treatment regimens from the 2023 ASH annual meeting. Figure 1 displays the protocols of selected studies, and Table 1 lists their outcomes.

**Table 1:** Chemotherapy-free regimens for adult patients with newly diagnosed Ph + ALL

Study Group or Author	No. of Pts	Age (ys)	CR/CRi	3 m CMR	Relapse	CNS relapse	Allo-SCT in CR1	Follow-up (ms)	OS	DFS or EFS	References
GIMEMA LAL2116 D-ALBA	63	54 (24–82)	98% (62/63)	29% (17/59)	15% (9/62)	6% (4/62)	39% (24/61)	53	80.7% at 4ys	75.8% at 4ys	[1]
BLISSPHALL	17	50 (22–87)	100% (17/17)	NA	12% (2/17)	0	23.5% (4/17)	11.7 (3–24)	All alive	NA	[2]
University of Colorado Hospital	14	54 (23–72)	100% (14/14)	NA	0	0	21% (3/14)	15 (4–24)	All alive	No relapse	[3]
GIMEMA ALLL2820	74	57 (20–80)	95% (55/58)	NA	2% (1/55)	0	0	6.1 (0−20.3)	NA	NA	[4]
MDACC Group	62	56 (20–83)	98% (39/40)	84% (46/55)	10% (6/61)	5% (3/61)	2% (1/61)	17 (2–61)	89% at 2ys	77% at 2ys	[5]
Zhang T, et al.	13	58/34.5	100% (13/13)	100% (13/13)	8% (1/13)	0	61.5% (8/13)	7	100% At 6ms	87.5% at 6ms	[6]
Gong XY, et al.	31	40 (20–66)	100% (31/31)	61.3% (19/31)	0	0	NA	5.8	All alive	No relapse	[7]
Xu GX, et al.	29	45 (19–74)	100% (25/25)	82.6% (19/23)	0	0	66.7% (16/25)	8	1 death	NA	[8]

Abbreviations: Pts, Patients; CR, Complete Remission; CRi, Complete remission with incomplete hematologic recovery; m, month; ms, months; CMR, Complete Molecular Remission; DFS; Disease-free Survival; EFS, Event-free Survival; CNS, Central Nervous System; Allo-SCT, allogeneic stem cell transplantation; CR1, First Complete remission; NA, not available.

## Blinatumomab-based treatment

Blinatumomab has gained attention for its effectiveness in clearing measurable residual disease (MRD). However, the optimal timing of its use has yet to be determined. The D-ALBA trial enrolled 63 newly diagnosed adult patients with Ph + ALL who received a combination of steroids and dasatinib for 85 days, followed by 2 to 5 cycles of blinatumomab and dasatinib consolidation and 12 doses of intrathecal (IT) chemotherapy [1]. During induction, 15 patients (24%) exhibited an increase in MRD during dasatinib monotherapy, 6 of whom had the *T315I* mutation and 1 of whom had the *E255K* mutation. Over a median follow-up of 53 months, nine relapses occurred—4 hematologic, 4 involving the central nervous system (CNS) and 1 nodal. Twenty-four (39%) patients underwent allogeneic stem cell transplantation (allo-SCT) in first complete remission (CR1). The estimated 4-year overall survival (OS) and disease-free survival rates were 80.7% and 75.8%, respectively. To expedite MRD clearance and suppress resistant clones early, the BLISSPHALL trial was designed to introduce blinatumomab as early as 6 weeks into the treatment of 17 patients with de novo Ph + ALL [2]. No patients showed an increase in MRD during induction. Four patients (24%) underwent allo-SCT in CR1. With a median follow-up of 11.7 months, two patients relapsed—1 with the *T315I* mutation who experienced extramedullary relapse—and no deaths occurred.

To suppress *T315I* clones and reduce recurrence in the CNS, several studies involving third-generation TKIs, adding chemotherapy, or increasing the IT chemotherapy dose are being designed. Jordan et al. explored the use of consolidation therapy comprising ponatinib plus blinatumomab (1–2 cycles) and chemotherapy which consisted of 4 cycles of HD-MTX/ID-Ara-C after dasatinib-based induction in 14 patients [3]. Three patients (21%) underwent allo-SCT in CR1. With a median follow-up of 15 months, no relapses or deaths occurred. In the experimental group of the GIMEMA ALL2820 trial (*n* = 58), dasatinib was replaced by ponatinib, which was given for 70 days, followed by ≥ 2 cycles of blinatumomab consolidation and 15 doses of IT chemotherapy [4]. The median follow-up was 6.1 months; only one patient, who was CD19 + and had the *T315I* mutation, relapsed. In the trial conducted by the MDACC Group (*n* = 62), patients received up to 5 cycles of blinatumomab with ponatinib, followed by 5 years of ponatinib and 12 IT chemotherapy doses. With a median follow-up of 17 months, six patients (10%) relapsed—two with hematological relapse (one with an *E225V* mutation), one with an extramedullary-only relapse, and three with a CNS-only relapse. Only 1 (3%) patient underwent allo-SCT in CR1, and the estimated 2-year event-free survival (EFS) and OS rates were 77% and 89%, respectively. In a study conducted by Ting Z et al., olverembatinib, a novel third-generation inhibitor, was used in combination with blinatumomab, followed by an additional cycle or allo-SCT (*n* = 13) [6]; eight (61.5%) patients underwent allo-SCT in CR1. With a median follow-up of 7 months, one patient with *E255V* relapsed after allo-SCT. The 6-month OS and EFS were 100% and 87.5%, respectively.

It’s noteworthy that the proportion of allo-SCT has significantly decreased in studies with ponatinib plus Blinatumomab, yet the survival of Ph + ALL patients remains unaffected or even improved. In the new era, the role allo-SCT as front-line treatment has been challenged, requiring more evidences to drawn definitive conclusion.

## Non-blinatumomab-based treatment

An approach currently being investigated by Chinese scholars incorporates venetoclax into a minimal chemo-based regimen [7, 8]. In the study conducted by Gong et al., thirty-one patients have received a combination of venetoclax, olverembatinib, vincristine, and prednisone for 28 days, followed by 2 cycles of venetoclax, olverembatinib and prednisone consolidation [7]. With a median follow-up time of 5.8 months, no relapses or deaths have occurred. Xu et al. designed a study based on the combination of olverembatinib, vindesine and prednisone (*n* = 29), in which patients will receive 3 cycles of this regimen [8]. Sixteen (67%) patients underwent allo-SCT in CR1. After a median follow-up of 241 days, one patient died, while all the remaining patients survived without relapse. An alternative novel regimen was the addition of CAR-T cell (CD19 and CD22) therapy to dasatinib/steroids/vincristine for newly diagnosed Ph + ALL in adults [9]. Eighteen patients were enrolled, and the CMR rate increased from 27.8% (5/18) after induction therapy to 72.2% (13/18) after CD19 CAR-T cell therapy, and increased further to 76.9% (10/13) after CD22 CAR-T cell therapy. No patient received allo-SCT. After a median follow-up of 13.5 months, 2 patients experienced relapses, and 14 of the remaining patients were in sustained CMR. The preliminary efficacy results have sparked our imagination in finding a chemo-free approach for Ph + ALL.

In this article, we review the impressive developments in chemo-free treatment strategies in Ph + ALL from ASH 2023. It important to note that the study design of references 3, 7, 8 and 9 involved the use of chemotherapy drugs (e.g., methotrexate and cytarabine consolidation, vincristine/vindesine), which may not entirely align with the chemo-free concept. However, the use of vincristine/vindesine was only in the first 1–3 courses of treatment, which are close to chemo-free concepts.

In conclusion, the ASH 2023 Annual Meeting demonstrated notable advances in the field of chemo-free therapy in adult patients with Ph + ALL, mainly focusing on the use of new targeted therapies to design an optimal regimen for improving outcomes.

## Data Availability

No datasets were generated or analysed during the current study.

## Abbreviations

Allo: SCT allogeneic stem cell transplantation
CNS: Central Nervous System
CR: Complete Remission
EFS: Event-free Survival
IT: Intrathecal
MRD: Measurable Residual Disease
OS: Overall Survival
TKI: Tyrosine Kinase Inhibitor
Ph + ALL: Ph-positive acute lymphoblastic leukemia
